# Across-province standardization and comparative analysis of time-to-care intervals for cancer

**DOI:** 10.1186/1471-2407-7-186

**Published:** 2007-10-04

**Authors:** Marcy Winget, Donna Turner, Jon Tonita, Charlotte King, Zoann Nugent, Riaz Alvi, Richard Barss

**Affiliations:** 1 Medical Affairs and Community Oncology, Alberta Cancer Board, 10123-99 Street, Edmonton, Alberta, T5J 3H1, Canada; 2 Epidemiology & Cancer Registry, CancerCare Manitoba, 675 McDermot Avenue, Winnipeg, Manitoba, R3E 0V9, Canada; 3 Saskatchewan Cancer Agency, 4101 Dewdney Avenue, Regina, Saskatchwan, S4T 7T1, Canada; 4 Saskatchewan Cancer Agency, 20 Campus Drive, University of Saskatchewan, Saskatchwan, S7N 4H4, Canada

## Abstract

**Background:**

A set of consistent, standardized definitions of intervals and populations on which to report across provinces is needed to inform the Provincial/Territorial Deputy Ministries of Health on progress of the Ten-Year Plan to Strengthen Health Care. The objectives of this project were to: 1) identify a set of criteria and variables needed to create comparable measures of important time-to-cancer-care intervals that could be applied across provinces and 2) use the measures to compare time-to-care across participating provinces for lung and colorectal cancer patients diagnosed in 2004.

**Methods:**

A broad-based group of stakeholders from each of the three participating cancer agencies was assembled to identify criteria for time-to-care intervals to standardize, evaluate possible intervals and their corresponding start and end time points, and finalize the selection of intervals to pursue. Inclusion/exclusion criteria were identified for the patient population and the selected time points to reduce potential selection bias. The provincial 2004 colorectal and lung cancer data were used to illustrate across-province comparisons for the selected time-to-care intervals.

**Results:**

Criteria identified as critical for time-to-care intervals and corresponding start and end points were: 1) relevant to patients, 2) relevant to clinical care, 3) unequivocally defined, and 4) currently captured consistently across cancer agencies. Time from diagnosis to first radiation or chemotherapy treatment and the smaller components, time from diagnosis to first consult with an oncologist and time from first consult to first radiation or chemotherapy treatment, were the only intervals that met all four criteria. Timeliness of care for the intervals evaluated was similar between the provinces for lung cancer patients but significant differences were found for colorectal cancer patients.

**Conclusion:**

We identified criteria important for selecting time-to-care intervals and appropriate inclusion criteria that were robust across the agencies that did not result in an overly selective sample of patients to be compared. Comparisons of data across three provinces of the selected time-to-care intervals identified several important differences related to treatment and access that require further attention. Expanding this collaboration across Canada would facilitate improvement of and equitable access to quality cancer care at a national level.

## Background

The Ten-Year Plan to Strengthen Health Care, established in September 2004 [1] by the Provincial/Territorial Deputy Ministries of Health, united the Premiers of all Canadian provinces through a national plan for healthcare in the next decade. Improving access to and timeliness of care were identified as high priority areas for improving the Canadian health care system. As part of an exchange for federal funding to the provincial health care system, every province is expected to report "wait times" in five priority areas, including cancer.

Each province has tackled this responsibility differently, resulting in a set of inconsistently-defined intervals, patient subsets, and metrics used to report "wait times" that vary across provinces. In the case of cancer care, for example, Alberta is monitoring time from referral to first visit with an oncologist and time from the first visit to treatment (for chemotherapy and radiation therapy separately) [2], while Manitoba is monitoring time from "ready to treat" to radiation therapy [3]. The health agencies of most provinces post "wait time" data online [2-7] in an attempt to be transparent and accountable to the public. Without a standardized reporting system, however, that unifies the definitions of care-step intervals to measure, patient populations to include, and metrics to report, it is not possible to properly evaluate progress across provinces for meeting the goals set forth in the Ten-Year Plan. Specifically, a set of consistent, standardized definitions of intervals and populations on which to report across provinces would allow provincial health-care agencies to discuss "wait times" in a unified manner, make across-province comparisons to identify policy-relevant strengths and deficiencies of each province in its care system, and learn from each other towards the achievement of the national Ten-Year Plan.

A large body of literature exists on issues associated with cancer care "wait times" nationally and internationally [8], however, the variability of methodologies used make comparison of results and use of information for decision-making difficult. In Canada, for example, there have been nine "wait-time" studies that measured time to care for colorectal and/or lung cancer patients [9-16]. All of them focused on measuring aspects of time to radiation therapy or time to surgery. Varying definitions of time points, study designs, patient populations and reporting metrics, however, make comparison of results impractical. Analyzing data on timeliness of care in a comparative fashion is a crucial research task for informing policy makers, health care providers, and other decision-makers towards improving the quality of patient care.

To address these concerns, researchers in the cancer agencies in three Canadian provinces (Alberta (AB), Saskatchewan (SK), and Manitoba (MB)) formed a collaborative team to develop and apply a unified methodology that is practical and useful in comparing and learning from each other, on time to cancer care in the three provinces. The cancer agencies in these provinces are responsible for non-surgical treatment of cancer in their jurisdictions. This manuscript describes the methodology developed from the collaboration and the overall analysis results as applied to invasive lung and colorectal cancer cases diagnosed in 2004 as the source population.

## Methods

To initiate the project, we identified and assembled a group of stakeholders from each of the three cancer agencies to participate in a workshop to identify steps that our cancer agencies could take towards standardizing definitions and measures of relevant cancer care intervals. At the beginning of the workshop, presentations were made to inform one another on current, planned, and future activities for measuring and reporting "wait times" at each of the cancer agencies for both internal and external purposes. Criteria to define "relevance" were then discussed and identified. Various intervals and corresponding start and end time points were suggested and evaluated with respect to the criteria. Final intervals to pursue were then selected that met the criteria and for which analysis and evaluation could be completed within the one-year time frame of the project were selected. The general consensus-forming process was as follows. Ideas were generated via brainstorming followed by open discussion, including identification of pros and cons. Ideas were then synthesized, discussed further, as needed, and then final priorities and directions were voted on.

The expertise amongst stakeholders in the workshop was broadly based and included senior management, epidemiologists, surgeons, oncologists, nurses, a biostatistician, data analysts, quality assurance experts, system developers, and programmers. Final decisions were made by group consensus via informal voting after extensive discussion in workshops. In addition to workshops attended by the entire collaboration, a core group held additional meetings to clarify the data elements available, processes for data extraction, data completeness and quality, and ensure comparability of data across the three cancer agencies.

Consideration of time-to-care measurements as indicators for timely access to care served as the underpinning for discussions and decisions around the criteria for time points and intervals. The following characteristics were identified as requirements for standardizing such indicators for cancer: 1) relevant to patients (as opposed to relevant for internal organizational management), 2) clinically relevant, 3) time points are unequivocally definable, and 4) practical in a sense that data for relevant time points are routinely collected at the provincial cancer agencies in a consistent and high quality manner.

Based on the four criteria, we evaluated potential start and end time points for relevant time intervals. The time points and intervals considered included those that were being used in one of the three provinces and/or had been proposed in the literature. This process led us to the final time points and corresponding intervals to be used in the project.

In addition to the definition and selection of time intervals, we also identified inclusion criteria for the patient population. We selected lung and colorectal cancers diagnosed in 2004 as exemplary tumor sites to be analyzed for the purpose of evaluating and comparing measures for the selected intervals. The reasons for this choice were: disease incidence is high for both, they affect men and women, and treatment includes the main cancer treatment modalities (surgery, radiation, and chemotherapy) [17]. The two cancers also contrast nicely in that prognosis is generally poor for lung cancer but good for colorectal cancer [18].

All invasive colorectal (ICD-0 codes [19] C18, C19, C20, C21, C260) and lung (ICD-0 codes [19] C33, C34) cancers diagnosed in 2004 in AB, SK, or MB that were of a "typical histology" (see Table 1 for list of excluded histologies) [17] and for which the patient had an "opportunity to receive treatment" were included in analyses; that is, the date of diagnosis was not concurrent with the date of death. In addition, dates of patient visits must be attributable specifically to the cancer diagnosis of interest. Currently, electronic scheduling systems in all three cancer agencies do not link a patient's diagnosis with their visits. We, therefore, excluded patients with another invasive [20] cancer diagnosis 183 days (6 months) prior to or after the diagnosis of interest.

**Table 1:** Sample selection criteria

	**Alberta**		**Saskatchewan**		**Manitoba**	
	**Colorectal**	**Lung**	**Colorectal**	**Lung**	**Colorectal**	**Lung**

	**N (%)**	**N (%)**	**N (%)**	**N (%)**	**N (%)**	**N (%)**

**Diagnosed^**1,2 **^in 2004**	1688 (100)	1672 (100)	743 (100)	636 (100)	849 (100)	895 (100)
**Exclusion criteria:**						
**Non-invasive**	74 (4)	2 (<1)	58 (8)	2 (<1)	36 (4)	15(2)
**Uncommon^**3 **^histology**	10 (<1)	5 (<1)	3 (<1)	1 (<1)	1 (<1)	1 (<1)
**Multiple^**4 **^cancers**	66 (4)	55 (3)	19 (3)	14 (3)	34 (4)	45 (5)
**Dead at diagnosis**	9 (<1)	17 (1)	21 (3)	52 (8)	17 (2)	34 (4)
**Final population:**	1529 (90)	1593 (95)	642 (86)	567 (89)	761 (90)	800 (89)

1- Diagnosis date defined by Canadian Cancer Registry coding rules [22(p67)]

2- Sites defined as in Canadian Cancer Statistics [16(p85)]

3- Leukemias, lymphomas, mesothelioma and Kaposi's sarcoma as defined in Canadian Cancer Statistics 2005 [16(p85)]

4- One or more invasive cancers within 6 months of the index case. Does not include non-melanoma skin cancer or insitu breast or bladder cancers as defined in [19(pp297-299)]

In addition to identifying inclusion criteria for patients, it was also necessary to identify inclusion criteria for dates of the endpoints of the intervals (e.g., diagnosis to treatment). Initially data were evaluated that included dates within 18 months of diagnosis. We found that 95% of the patients received their first treatment in a cancer facility within 6 months. The remaining patients either had watchful waiting as their initial treatment plan or refused the recommended first treatment. Upon investigation of the data, we found that a very small proportion of patients were meeting with an oncologist or receiving treatment prior to diagnosis. This was a result of patients being referred prior to having a definitive diagnosis or errors in coding that have since been corrected. We, therefore, included dates of visits in the range between 30 days prior to diagnosis to 183 days (6 months) post-diagnosis.

The final dataset was reviewed by each province and quality assurance checks were run to ensure accuracy and completeness. Descriptive statistics and inverse Kaplan-Meier [21] graphs were generated for each interval of interest; the Log-Rank [22] test was used to test for differences between curves. Estimates for the 50th and 90th percentiles and their corresponding 95% confidence intervals were calculated non-parametrically. Chi-squared tests [22] were used to test differences between proportions. SAS^® ^version 9.1 (Cary, NC, USA) was used to create the datasets and analyze them; graphs were made using Microsoft Excel^®^.

## Results

Table 2 describes the three Canadian provinces and their respective cancer agencies. The cancer agencies are organizationally and functionally similar and serve respective provincial populations that are comparable.

**Table 2:** Description of provinces and corresponding cancer agencies

**Province and cancer agency facts**	**Alberta**	**Saskatchewan**	**Manitoba**
**Province:**			
Land area:	661,185 sq. km	651,900 sq. km	649,950 sq. km
Population:	3.1 million	1.0 million	1.2 million
Annual cancer incidence^1^:	12,000	5,000	5,600
**Cancer Agency: **# of cancer facilities^2 ^that provide:			
Radiation and chemotherapy:	2	2	1
Chemotherapy only:	14	13	14
Surgery:	0	0	0
Diagnostic imaging:	1	0	0

1- Exlcuding non-melanoma skin cancer

2- Facilities that are within the organizational structure of the cancer agency

The time points in a cancer patient care-trajectory that were considered and their relationship to the four criteria, described in the *Methods*, are summarized in Table 3. Only four time points met all the criteria: dates of diagnosis, first consult with an oncologist, first radiation, and first chemotherapy. Date of diagnosis is captured by each province's cancer registry according to the Canadian Cancer Registry coding rules [23], which define it as the earlier of the date of the test for which the first pathological evidence of cancer is available or the date of the first test on which the treatment plan is based. The latter date is used if the patient receives cancer treatment prior to or in the absence of pathological evidence. Dates of first consult with an oncologist, radiation treatment, and chemotherapy are captured in each province's electronic medical record system as part of their scheduling procedures. Treatment dates are captured by both the cancer registry (retrospectively) and by the cancer electronic medical records (in real time).

**Table 3:** Time points and criteria for selecting standardized time-to-care intervals

**Time points**	**Criteria**			
Date of first:	Relevant to patients	Relevant to clinical care	Unequivocally defined	Currently captured^1^

Symptom onset	Yes	Yes	No	No
Diagnosis	Yes	Yes	Yes	Yes
Referral to cancer facility	Consensus not reached^2^	Consensus not reached^2^	No	No
Consult with oncologist	Yes	Yes	Yes	Yes
Ready to treat	No	Consensus not reached^2^	No	No
Surgery	Yes	Yes	Yes	No
Radiation	Yes	Yes	Yes	Yes
Chemotherapy	Yes	Yes	Yes	Yes

1 – If time point is currently captured routinely and consistently by the cancer agencies of AB, SK, and MB then 'Yes' otherwise 'No.'

2 – Consensus regarding the relevance of the time point was not reached by the participants in the workshop in the process described in the *Methods*.

We considered three intervals with the four time points that satisfied the criteria; they were, time from: 1) diagnosis to first treatment in a cancer facility (that is, radiation or chemotherapy), 2) diagnosis to first consult with an oncologist, and 3) first consult with an oncologist to first treatment in a cancer facility. Interval (1), above, was selected as the primary interval of interest for comparison because, of the three intervals, it is the best summary measure of the total time-to-care experience of patients post-diagnosis. Times from diagnosis to first consult and from first consult to first radiation/chemotherapy were agreed to be important components of the primary interval. The provincial 2004 colorectal and lung cancer data are used to illustrate across-province comparisons.

Table 1 lists the total number of cases diagnosed in 2004 and the number excluded by each province for each exclusion criterion. Depending on the cancer site and province, between 86% and 95% of the original sample was retained after the exclusion criteria were applied. Table 4 lists the distribution of demographic characteristics of the cases included in the final population by province and cancer site (lung/colorectal cancer). The age and gender distribution is similar across provinces and cancer sites. The proportion of urban cases in each province is comparable to the proportion of urban residents in each province.

**Table 4:** Distribution (percent) of patient demographics of final population

	**Colorectal**			**Lung**		
	**Alberta (N = 1518)**	**SK (N = 642)**	**Manitoba (N = 749)**	**Alberta (N = 1585)**	**SK (N = 567)**	**Manitoba (N = 784)**

**Age**^**1**^						
<=69	47	43	46	51	46	46
70–79	31	34	31	33	36	34
>=80	22	23	24	15	18	20
**Gender**						
Female	44	46	47	47	46	48
Male	56	54	53	53	54	52
**Region**^**2**^						
Urban	62	50	55	63	50	57
Rural	38	50	45	37	50	43

1 -Percents may not add to 100 due to rounding

2 -Urban defined as living in a health region with major cancer facility (i.e., health regions containing the cities of Edmonton, Calgary, Regina, Saskatoon, or Winnipeg) at time of diagnosis.

**Figure 1:**
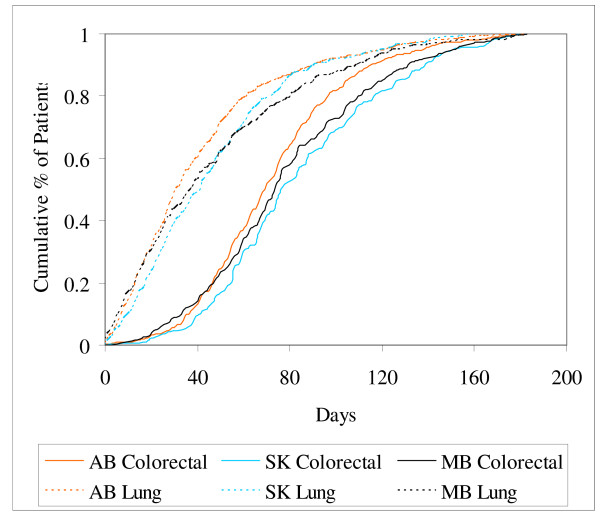
Cumulative time from diagnosis to first radiation or chemotherapy treatment by tumor site and province.

Figure 1 shows the cumulative proportion of patients who received radiation or chemotherapy by the time elapsed in days from diagnosis. The time interval is much shorter for lung cancer patients than colorectal cancer patients. The time from diagnosis to first radiation/chemotherapy is significantly different across the provinces for both colorectal (P = 0.002) and lung cancer patients (P = .0061).

**Figure 2:**
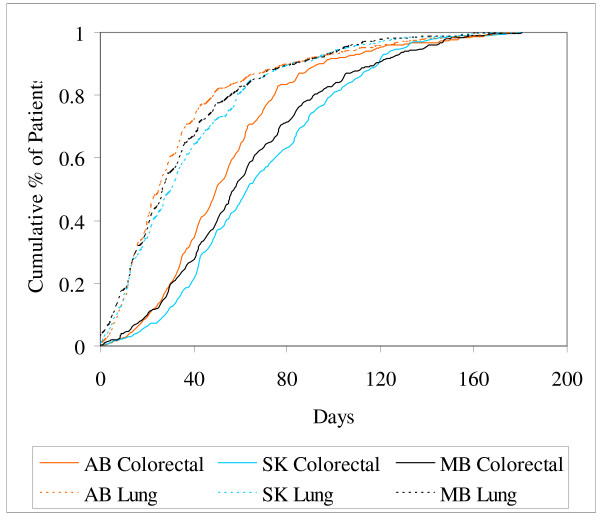
Cumulative time from diagnosis to first consult visit with an oncologist by tumor site and province.

**Figure 3:**
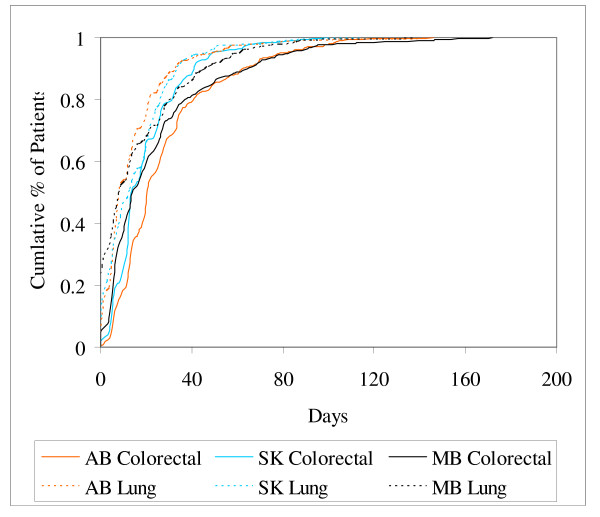
Cumulative time from first consult visit with an oncologist to first radiation or chemotherapy treatment by tumor site and province.

The curves shown in Figures 2 and 3 present the time from diagnosis to first consult with an oncologist and time from first consult to first radiation/chemotherapy, respectively. From Figure 2 it can be seen that the time from diagnosis to first consult was much shorter for lung cancer patients than for colorectal cancer patients. In Figure 3, however, the difference between tumor sites is negligible indicating a similarity in speed of processing and treating patients once they are seen at a cancer facility by an oncologist. The time intervals from diagnosis to consult and consult to radiation/chemotherapy for colorectal cancer patients were both significantly different across provinces (P < 0.001 for both intervals). Colorectal cancer patients in AB experienced a shorter time from diagnosis to consult with an oncologist but a longer interval from consult to radiation/chemotherapy than in SK or MB. No differences were found in either interval for lung cancer, (P = 0.29 and P = 0.17, respectively).

Table 5 summarizes the information in Figures 1, 2, 3 using the 50th and 90th percentiles of time to event in days and their corresponding 95% confidence intervals, by province and tumor site. Confidence intervals tend to be fairly wide for all intervals. Although statistical differences identified graphically and in Table 5, based on significance testing and overlapping confidence intervals, respectively, are fairly consistent with one another, conclusions are likely to be different depending on the measure chosen for comparisons.

**Table 5:** Median and 90^th ^percentile estimates for time-to-care for each care interval by cancer site and province

	**# days from diagnosis to 1^**st **^radiation or chemotherapy**		**# days from diagnosis to consult**		**# days from consult to 1^**st **^radiation or chemotherapy**	
	
	**Median (95% CI)**	**90% (95% CI)**	**Median (95% CI)**	**90% (95% CI)**	**Median (95% CI)**	**90% (95% CI)**
**Colorectal**						
**Alberta**	69 (67, 74)	115 (110, 124)	50 (48, 53)	95 (89, 104)	21 (20, 21)	62 (56, 70)
**Saskatchewan**	77 (73, 85)	139 (132, 150)	63 (59, 69)	120 (113, 122)	14 (13, 18)	41 (36, 48)
**Manitoba**	74 (70, 77)	132 (122, 144)	57 (54, 60)	118 (107, 127)	14 (12, 19)	64 (51, 77)
**Lung**						
**Alberta**	31 (29, 34)	91 (84, 99)	25 (23, 26)	81 (72, 90)	8 (8, 9)	33 (30, 35)
**Saskatchewan**	41 (35, 45)	91 (80, 112)	30 (27, 33)	82 (71, 99)	10 (8, 14)	34 (30, 41)
**Manitoba**	36 (29, 41)	109 (95, 119)	25 (22, 28)	78 (70, 95)	8 (7, 12)	46 (41, 55)

## Discussion

The main goals of this project were to: 1) identify a set of criteria and variables needed to create comparable measures of important time-to-cancer-care intervals that could be applied across provinces and 2) to use the measures to compare time-to-care across participating provinces for lung and colorectal cancer patients diagnosed in 2004. Extensive communication and inclusion of a broad set of expertise ensured that the various perspectives of different types of stakeholders were included in the consensus-reaching process. This approach was needed to identify appropriate inclusion criteria that were robust across the agencies and did not result in an overly selective, or possibly biased, sample of patients to be compared. Application of the method to both lung and colorectal cancer patients provided examples for different types of cancer with different standard treatment practices.

Differences observed between provinces in the intervals depicted in Figures 2 and 3 for colorectal cancer patients suggest each cancer agency could learn from another to shorten the intermediary care intervals and subsequently shorten the overall diagnosis to first radiation/chemotherapy interval. Differences in time-to-care could be due to differences across provinces in the distribution of patient/disease charateristics, referral patterns, and/or treatment patterns, in addition to care-delivery differences. Patient characteristics were, however, comparable across the provinces (see Table 4). The distribution of stage at diagnosis for colorectal cancer patients across the provinces was also comparable (data not shown). Further investigations into referral patterns and treatment practices in the three provinces are underway to understand and explain the differences seen.

In Figures 1 and 2, the differences between lung and colorectal cancer patients are largely due to differences in treatment practice. Roughly 85% of the colorectal cancer patients had surgery prior to radiation or chemotherapy, whereas only about 13% of the lung cancer patients did. Surgery is more likely to be a colorectal cancer patient's first treatment while radiation or chemotherapy is more likely to be a lung cancer patient's first treatment. To a large extent then, these graphs represent time to first and second treatment for lung and colorectal cancer patients, respectively. Comparable differences would be expected to be seen between any tumor sites with similarly different order in their treatment modalities.

Ideally, one would like to compare time from diagnosis to first treatment and time from first treatment to second treatment. This, however, is not currently possible at a national level in Canada because a major treatment modality, surgery, is performed outside of the cancer agencies and many cancer agencies do not have routine access to cancer surgery data. Until dates for treatments received outside cancer agencies are routinely available, however, reporting time from diagnosis to first treatment oferred at the cancer agency is practical; at the very least it should be comparable for most Canadian cancer agencies. Based on an unpublished survey conducted by the Candian Association of Provincial Cancer Agencies (CAPCA) in 2006 at least seven provinces in Canada have the dates of diagnosis, consult with oncologist and first treatment in a cancer facility; the other three provinces may not have dates related to chemotherapy readily available (this information was not specifically captured in the survey) but have diagnosis date and dates related to radiation (consultations and treatment).

The Health Council of Canada recommends the definition of a waiting interval be time from initial referral to completion of the procedure [24]. "Initial referral," in the context of cancer care, however, can have several meanings since patients may have multiple "initial" referrals as they are referred to surgeons and various types of oncologists. The clock of "waiting," however, begins at the time of diagnosis for the patient, thus diagnosis is a more appropriate start time. Furthermore, delays that occur between diagnosis and any of these "initial" referrals may be clinically relevant to quality of patient care. Regarding reporting, the Health Council suggests reporting the duration in 90^th ^or 95^th ^percentiles and adds that a wait-time reporting system should inform on the time for typical cases relative to existing benchmarks, duration of interval for outliers, and changes over time. Information on time-to-care alone is not enough, however, to properly inform policy on quality of care for cancer. Additional information such as the proportion of patients receiving certain care steps is also needed; short time-to-care intervals do not equal high quality if a large percentage of patients who should be receiving care are not.

An important limitation in the process used to identify criteria and select relevant time intervals was that we had one year of funding to perform the work; this prevented us from considering time points that were not currently being collected by our cancer agencies or pursuing external data sources. This restriction also prevented us from considering points of care that fall outside the care of the cancer agencies such as pre-diagnosis and post-treatment follow-up; equally important intervals of care for which information related to timeliness of care is needed. Due to the limited timeframe, our intent was to identify an initial step towards standardizing defintions and measures related to timeliness of care that would facilitate future decision-making and identify directions that would eventually include the cancer care continuum. In this intial step we did not include patients in our decision-making process which is also a limitation. A limitation in interpreting the analytic results is that although differences between tumor sites and provinces can be detected, additional data and information are needed to identify why differences exist.

## Conclusion

In summary, production of standardized "wait-time" data across Canadian provinces will allow identification at the national level of problems which facilitate solutions and changes to current resource allocation and overall system improvements. Interpretation of the data in the context of practice guidelines, particularly as related to treatment modality and order, is essential to avoid incorrect interpretation of apparant relative delays. Production of standardized "wait-time" data requires close collaboration, at least initially, between the provinces to ensure comparability. This activity in and of itself may facilitate adoption of more efficient processes or models of care as differences and strengths between provinces are identified. Infrastructure must be developed to obtain dates of cancer surgery to enable comparisons of diagnosis to first and second treatment as well as facilitate evaluation of appropriate care.

Although there are many similarities between AB, SK, and MB with respect to the size of the provinces and organization of the cancer agencies, this collaboration has enabled us to identify several important differences related to treatment and access that require further attention. We expect that there are changes each organization could make that would decrease these differences resulting in improved access and quality of care for cancer patients. Expanding this collaboration across Canada would facilitate improvement of and equitable access to quality cancer care at a national level and reaching the goal of the Ten-Year Plan to Strengthen Health Care in Canada.

## Competing interests

The author(s) declare that they have no competing interests.

## Authors' contributions

MW conceived and designed the study, oversaw analyses, assisted with interpretation of the data, drafted the manuscript, and supervised the study. DT and JT conceived and designed the study, oversaw analyses, assisted with interpretation of the data and manuscript revision. RA conducted data analysis and assisted with study design, interpretation of the data, and manuscript revision. ZN performed data acquisition and data analysis, assisted with study design, data interpretation and manuscript revision. CK performed data acquisition and data analysis, assisted with data interpretation and manuscript revision, and provided administrative and technical support. RB performed data acquisition and assisted with data interpretation and manuscript revision. All the authors have read and approved the final version of the manuscript.

## Pre-publication history

The pre-publication history for this paper can be accessed here:


